# Organic Ligands Armored ZnO Enhances Efficiency and Stability of CsPbI_2_Br Perovskite Solar Cells

**DOI:** 10.1002/advs.202000421

**Published:** 2020-09-27

**Authors:** Pang Wang, Hui Wang, Yuchao Mao, Huijun Zhang, Fanghao Ye, Dan Liu, Tao Wang

**Affiliations:** ^1^ School of Materials Science and Engineering Wuhan University of Technology Wuhan 430070 China; ^2^ State Key Laboratory of Silicate Materials for Architectures Wuhan University of Technology Wuhan 430070 China

**Keywords:** high efficiency, inorganic perovskite solar cells, organic ligands, photostability, zinc oxide

## Abstract

Inorganic perovskite solar cells (PSCs) have witnessed great progress in recent years due to their superior thermal stability. As a representative, CsPbI_2_Br is attracting considerable attention as it can balance the high efficiency of CsPbI_3_ and the stability of CsPbBr_3_. However, most research employs doped charge transport materials or applies bilayer transport layers to obtain decent performance, which vastly complicates the fabrication process and scarcely satisfies the commercial production requirement. In this work, all‐layer‐doping‐free inorganic CsPbI_2_Br PSCs using organic ligands armored ZnO as the electron transport materials achieve an encouraging performance of 16.84%, which is one of the highest efficiencies among published works. Meanwhile, both the ZnO‐based CsPbI_2_Br film and device show superior photostability under continuous white light‐emitting diode illumination and improved thermal stability under 85 °C. The remarkable enhanced performance arises from the favorable organic ligands (acetate ions) residue in the ZnO film, which not only can conduce to maintain high crystallinity of perovskite, but also passivate traps at the interface through cesium/acetate interactions, thus suppressing the photo‐ and thermal‐ induced perovskite degradation.

## Graphical abstract

An inorganic CsPbI_2_Br perovskite solar cell employing organic ligands armored ZnO as the electron transport materials achieves a maximum power conversion efficiency of 16.84%, with superior photo‐ and thermal‐ stabilities.

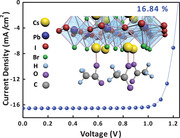

## 1 Introduction

Perovskite optoelectronic devices have skyrocketed tremendous progress over the past decade. As one of most significant applications, hybrid organic–inorganic perovskite solar cells (PSCs) have achieved 25.2% certified power conversion efficiency (PCE) due to the excellent optoelectronic properties of perovskite semiconductors.^[^ ^1^ ^]^ However, the instability of MA^+^ and FA^+^ (MA = methylamine, FA = formamidine) organic cations of hybrid perovskite impedes the commercial process of PSCs as the photovoltaic modules require high thermal stability during long‐term solar irradiation. Thus, recent researches have introduced alkali metal cesium Cs^+^ into the crystal lattice to stabilize the perovskite structure,^[^ ^2^, ^3^, ^4^ ^]^ however this can only alleviate rather than eliminate the thermal instability. Therefore, all inorganic perovskites are introduced to suffer extreme thermal condition, which can hold stable perovskite phase over 300 °C.^[^ ^5^, ^6^ ^]^

CsPbBr_3_ is a very stable perovskite material, PSCs of which have demonstrated nearly no degradation under 90−95% relative humidity (RH) or 100 °C over 800 h, but the wide bandgap (≈2.3 eV) of CsPbBr_3_ makes it hardly to absorb sunlight over 550 nm, thus achieving unsatisfied photovoltaic performance.^[^ ^7^ ^]^ Though CsPbI_3_ with a bandgap of ≈1.73 eV can extend the absorption spectrum to 720 nm to receive enhanced current density and PCE, the heavily deflected Goldschmidt tolerance factor (0.81) of CsPbI_3_ make it easy to form undesired non‐perovskite *δ*‐phase from *α*‐phase in ambient atmosphere, even in glovebox.^[^ ^8^ ^]^ Considering adding smaller radius Br^−^ into the I‐based perovskite film can improve phase stability, which have been widely employed in organic‐inorganic hybrid PSCs, many researches have attempted to change the ratio of I^−^ and Br^−^ of inorganic PSCs to balance performance and stability.^[^ ^9^, ^10^, ^11^, ^12^ ^]^ As a prominent representative, CsPbI_2_Br, with reduced phase transition temperature and desired bandgap (≈1.92 eV) between CsPbBr_3_ and CsPbI_3_, perform decent PCE and stability by various methods since 2016.^[^ ^12^, ^13^, ^14^, ^15^, ^16^, ^17^, ^18^, ^19^ ^]^ Hayase and co‐workers reinforced the phase stability and performance of PSC by partially substituting Pb with germanium (Ge) to form CsPb_0.8_Ge_0.2_I_2_Br, presenting excellent stability in ambient atmosphere.^[^ ^13^ ^]^ Li and co‐workers combined gradient thermal annealing and green anti‐solvent treatment to precisely control the morphology of CsPbI_2_Br, obtaining a high PCE of 16.07% and excellent ultraviolet (UV) stability in N_2_ atmosphere.^[^ ^14^ ^]^ Cao and co‐workers employed dual interfacial organic molecules to passivate trap state and restrain the photo‐induced halide segregation of CsPbI_2_Br, whilst enhancing the PCE up to 16.2% and the long‐term photostability.^[^ ^15^ ^]^ Yang and co‐workers used oleylammonium to induce the secondary grain growth of perovskite film, enlarging grain sizes up to 4 µm and maintaining 90% of PCE under 85 °C after 500 h.^[^ ^16^ ^]^

For the aforementioned attempts to achieve high performance and stability, most of them employ titanium dioxide (TiO_2_) or tin dioxide (SnO_2_) as the electron transport materials (ETMs), which is not as ideal as zinc oxide (ZnO) due to the more matched energy level of ZnO with perovskite, as well as the higher electron mobility and transparency of ZnO ETM comparing to TiO_2_ and SnO_2_.^[^ ^20^, ^21^ ^]^ However, ZnO rarely attracts the attention of researchers in PSCs though it has been widely applied in organic solar cells,^[^ ^22^, ^23^, ^24^ ^]^ because ZnO can deprotonate MA^+^ and FA^+^ cations and therefore induce the degradation of hybrid perovskites. Although incorporating inorganic cations (potassium, rubidium, Cs^+^) into the organic/inorganic hybrid perovskites or pretreating ZnO materials can stabilize perovskite to some extent, the unsatisfied stability and complicated fabrication processes are not favorable during commercialization.^[^ ^25^, ^26^, ^27^, ^28^ ^]^ After substituting all organic cations with Cs^+^ to form inorganic perovskite, organic components are absent to allow ZnO to degrade. Cao and co‐workers^[^ ^29^ ^]^ applied a SnO_2_/ZnO bilayer ETL to promote desirable cascade energy levels with CsPbI_2_Br, devices of which exhibit improved stability and open‐circuit voltage (*V*_OC_) (1.06 to 1.23 V), demonstrating that ZnO is a promising ETM for inorganic PSCs. Besides, state‐of‐the‐art inorganic PSCs utilized doped HTMs (Spiro‐OMeTAD or PTAA) or bilayer ETMs to obtain high efficiency, however the introduction of dopants will induce the fast degradation of devices and the bilayer ETMs will complicate the fabrication process.^[^ ^14^, ^15^, ^16^, ^30^, ^31^ ^]^ Therefore, it is urgent to demonstrate high‐efficiency and stable inorganic PSCs with simplified preparation procedures.

Herein, we have fabricated all‐layer‐doping‐free planar heterojunction inorganic PSCs with a structure of Glass/ITO/ETM/CsPbI_2_Br/polyTPD/MoO_3_/Ag by employing organic ligands armored ZnO as the ETM, which remarkably outperform the TiO_2_‐ and SnO_2_‐ based devices. After optimizing thermal annealing temperatures to control the content of organic ligands, champion device of ZnO‐based inorganic PSCs exhibits a high PCE of 16.84%, which is one of the highest photovoltaic performance of CsPbI_2_Br‐based inorganic PSCs. Upon a continuous white light LED illumination, ZnO/perovskite film can maintain integrity over 100 h in ≈10% RH and the ZnO‐based device with encapsulation can maintain 90% PCE over 200 h. Besides, the ZnO‐based device can maintain 86% PCE after 400 h continuous thermal annealing under 85 °C. We attribute the enhanced performance, photostability and thermal stability to the proper content of acetate ions in the ZnO film, which can not only conduce to maintain excellent crystallinity of perovskite but also passivate perovskite film at the interface through cesium/acetate interactions that were confirmed through the comprehensive analysis of XPS, FTIR, ^1^H NMR and theoretical calculation. Our work provides a promising ETM candidate and facile procedure to fabricate high performance and stable inorganic PSCs.

## 2 Results and Discussion

**Figure 1:**
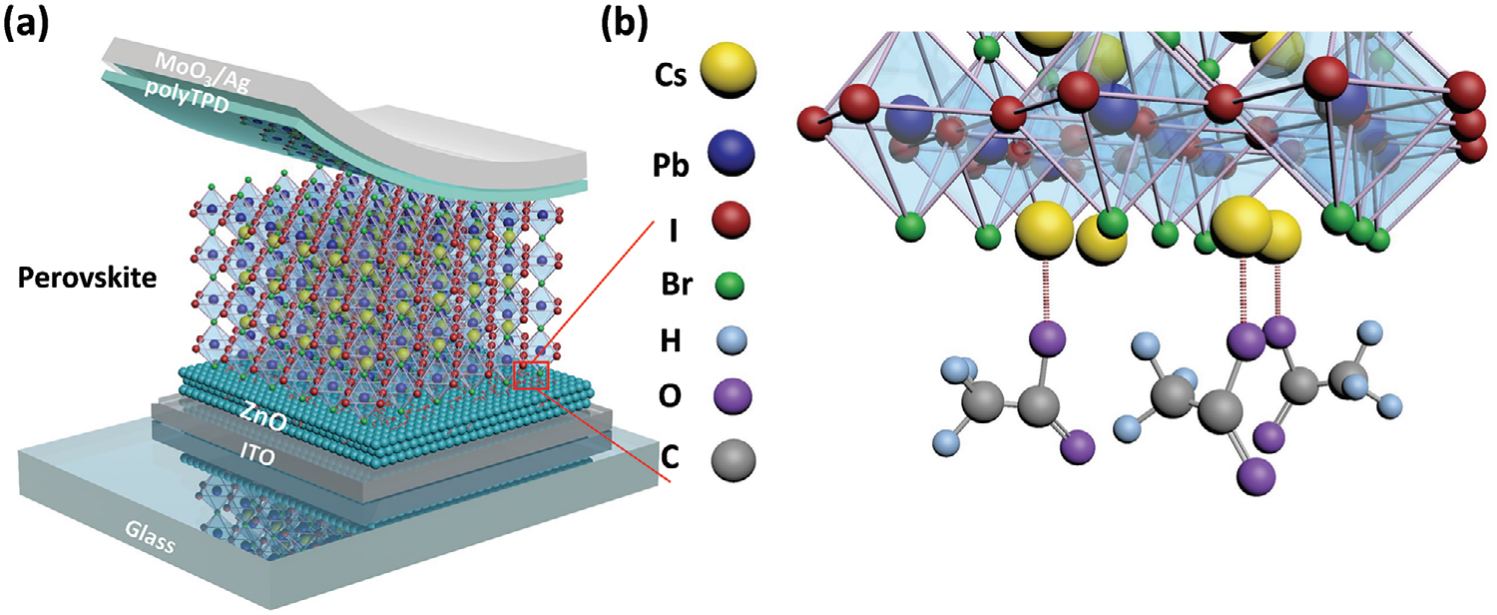
a) Structure of an n–i–p type inorganic PSCs. b) Scheme of interactions between CH_3_COO^−^ ligands and CsPbI_2_Br.

We employed a two‐step annealing process to fabricate smooth perovskite film using TiO_2_, SnO_2_, and ZnO as ETMs, with the ZnO based device structure was shown in **Figure** **1**a. As recorded in Figure S1 (Supporting Information), we measured the performance of inorganic PSCs using different ETMs under AM 1.5G simulated sun light, and obtained improved PCE from 11.6% (TiO_2_) and 12.4% (SnO_2_) to 16.84% (ZnO) from reverse scans, derived from the elevated *V*_OC_ (from 1.01 and 1.09 to 1.24 V), short‐circuit current (*J*_SC_) (from 15.2 and 15.0 to 16.5 mA cm^−2^) and fill factor (FF) (from 75.5% and 75.8% to 82.1%). Admittedly, there are hysteresis in all these PSCs, agreeing well with literature reports.^[^ ^16^, ^30^, ^32^, ^33^, ^34^ ^]^ The enhanced *V*_OC_ arises from more matched energy level alignment between ZnO (−4.31 eV) and CsPbI_2_Br (−4.16 eV) compared to TiO_2_ (−4.43 eV) (Figure S1a, Supporting Information), which is conducive to decrease energy barrier and facilitate electron transport at the ETMs/perovskite interface.^[^ ^29^, ^35^ ^]^ Besides the quality of the perovskite layer grown on these ETMs and the interfacial charge transport efficiency, the higher electron mobility and transparency of ZnO also contribute to promote *J*_SC_ and FF, which have been widely reported in many publications.^[^ ^20^, ^36^, ^37^ ^]^ The performance of those TiO_2_ and SnO_2_ based PSCs can be improved upon further bulk and interfacial treatments.^[^ ^18^ ^]^ Afterward, SnO_2_/CsPbI_2_Br and ZnO/CsPbI_2_Br films were selected to be illuminated under simulated sunlight at 20–30% RH to evaluate the photostability of perovskite films, and the corresponding evolution of measured PL were recorded in Figure S2a,b, Supporting Information. The continuous increase of PL intensity and emission wavelength in SnO_2_/CsPbI_2_Br and ZnO/CsPbI_2_Br films indicate photo‐induced halide phase separation of CsPbI_2_Br, which has been widely observed in mix‐halide PSCs, and the ambient humidity (20–30% RH) may amplify the evolution process.^[^ ^38^, ^39^, ^40^, ^41^, ^42^ ^]^ However the emission wavelength of ZnO/CsPbI_2_Br film only increases from 646 to 653 nm, which is much lower than the SnO_2_/CsPbI_2_Br film (646 to 663 nm) (Figure S2c, Supporting Information), suggesting that the ZnO film can effectively slowdown the halide phase separation of CsPbI_2_Br therefore contribute to enhanced photostability.

**Figure 2:**
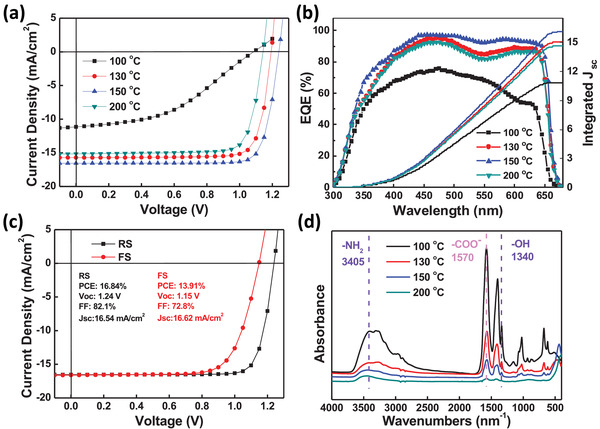
a) *J*–*V* curves of inorganic CsPbI_2_Br PSCs with different temperature annealing ZnO. b) EQE spectra and corresponding integrated current densities. c) *J*–*V* curves of champion device with reverse and forward scans. d) Infrared adsorption spectrum of ZnO films, all films are spin coating on KBr pellets and annealed on hotplate.

In order to clarify the reasons of the simultaneously enhanced performance and photostability of ZnO‐based device, we altered the ZnO annealing temperature to obtain different performance of devices and the corresponding device *J*–*V* curves from reverse scans are illustrated in **Figure** **2**a. The device with 100 °C annealed ZnO gives an unsatisfied performance of 5.91%, a *V*_OC_ of 1.08 V, a *J*_SC_ of 11.14 mA cm^−2^ and an FF of 43.1%. After elevating the annealing temperature toward ZnO, device PCE first increases to 15.32% and 16.84% upon annealing at 130 and 150 °C then decreases to 13.84% upon annealing at 200 °C, and all photovoltaic parameters vary with PCEs including *V*_OC_ (1.19, 1.24, and 1.14 V), *J*_SC_ (15.72, 16.54, and 15.16 mA cm^−2^) and FF (81.7%, 82.1%, and 79.9%). Figure 2b illustrates the external quantum efficiency (EQE) of four kinds of devices, and the relevant integrated *J*_SC_ values are 10.80, 15.02, 16.06, and 14.62 mA cm^−2^ respectively. The difference of *J*_SC_ obtained from *J*–*V* curves and EQE are all below 5%, confirming the accuracy of current density from *J*–*V* curves. The detailed photovoltaic parameters are summarized in **Table** **1**. Figure 2c shows the *J*–*V* curves of champion device from the reverse scan with a PCE of 16.84% and from the forward scan with a PCE of 13.91%, which is the highest performance in inorganic perovskite based on CsPbI_2_Br devices (see summary in Table S2, Supporting Information). Admittedly, the hysteresis of CsPbI_2_Br remains outstanding and urges further investigation via bulk or interfacial modifications to reduce defect and balance charge extraction.

**Table 1:** Photovoltaic parameters of CsPbI_2_Br PSCs obtained from reverse scans employing ZnO ETM annealed at different temperatures

Temperature [°C]	*V*_OC_ [V]	*J*_SC_ [mA cm^−2^]	Calculated *J*_SC_ [mA cm^−2^]	FF [%]	PCE_max_ [%]	PCE_ave_ [%]^a)^
100	1.08	11.14	10.80	43.1	5.91	4.85 ± 1.05
130	1.19	15.72	15.02	81.7	15.32	14.58 ± 1.47
150	1.24	16.54	16.06	82.1	16.84	15.90 ± 1.21
200	1.14	15.16	14.62	79.9	13.84	13.02 ± 0.97

^a)^ The PCE_ave_ ± error bar was obtained based on 20 individual devices.

We continued to measure the transmittance and X‐ray diffraction (XRD) of ZnO films annealed at 100, 130, 150, and 200 °C, and find that there are no obvious differences,^[^ ^20^ ^]^ meantime the conductivity (3.58 × 10^−3^, 3.93 × 10^−3^, 4.00 × 10^−3^, and 3.49 × 10^−3^ mS cm^−1^) and electron mobility (4.39 × 10^−3^, 4.36 × 10^−3^, 4.10 × 10^−3^, and 4.05 × 10^−3^ cm^2^ V^−1^ s^−1^) are also similar, so the variations of efficiency are not strongly related to these parameters of the ZnO film (Figure S3, Supporting Information). Then we proceeded to deposit ZnO with the same thickness on KBr pellets to measure the infrared absorption spectrum after annealing at 100, 130, 150, and 200 °C, therefore we can probe the relative amount of organic ligands by comparing the absorption intensity. As shown in Figure 2d, the peaks at 3405 (–NH_2_) and 1340 (–OH) nm^−1^ can represent ethanolamine, 1570 (COO^−^) nm^−1^ can represent CH_3_COO^−^ (Ac^−^).^[^ ^43^ ^]^ With the increase of annealing temperature, the amount of organic compounds in ZnO thin films decreases dramatically, indicating less organic ligands could interact with perovskite. Thereafter, we used scanning electron microscope (SEM) to observe the morphology of perovskites deposited on different temperature annealed ZnO. In Figure S4a–d, Supporting Information, perovskite cast on 100 °C annealed ZnO exhibits lots of pinholes, probably arising from the secondary volatilization of organic ligands during the perovskite crystallization process (annealing at 240 °C). After improving the annealing temperature of ZnO, pinholes of perovskite films are obviously reduced by casting on ZnO annealed at 130 °C then completely eliminated by casting on ZnO annealed at 150 and 200 °C. Therefore less organic ligands of ZnO guarantees the formation of smooth and compact perovskite films, which can tremendously reduce pinhole‐induced film defects. The XRD spectra of perovskite films grown on these ZnO are shown in Figure S4e (Supporting Information), from which we can identify stronger diffraction peaks of perovskite cast on ZnO annealed at 150 °C, which corresponds to higher crystallinity. The enhanced crystallinity of CsPbI_2_Br perovskite will improve the light absorption ability and lead to higher *J*_SC_, a result that is consistent with our *J*–*V* measurements presented in Figure 2a and Table 1.

**Figure 3:**
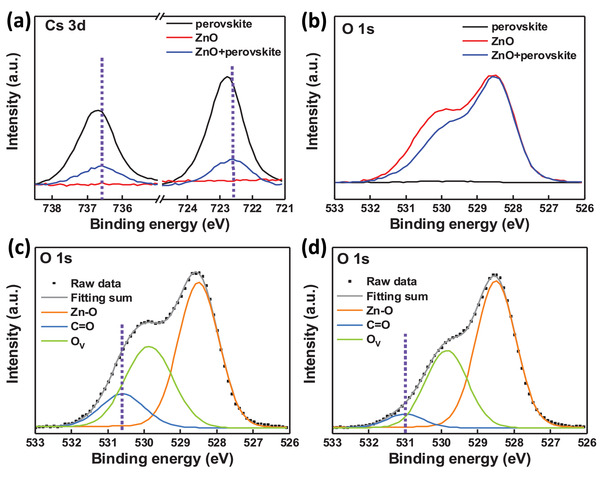
XPS spectra of perovskite, ZnO and ZnO/perovskite films of a) Cs 3d and b) O 1s. O 1s fitting data of c) ZnO and d) ZnO/perovskite films.

X‐ray photoelectron spectroscopy (XPS) is a powerful tool to probe the interaction between different thin films.^[^ ^15^, ^44^, ^45^ ^]^ Hence we fabricated films of CsPbI_2_Br, ZnO and ZnO/CsPbI_2_Br to investigate interactions between organic ligands and CsPbI_2_Br. **Figure** **3**a,b shows the Cs 3d and O 1s data of CsPbI_2_Br, ZnO, and ZnO/CsPbI_2_Br films. After the deposition of an ultrathin CsPbI_2_Br film (≈7 nm) on ZnO, the Cs 3d peak shifted toward a lower binding energy compared to the pristine CsPbI_2_Br film, indicating that electron density around Cs atoms is increased (Figure 3a). Meantime O 1s of ZnO and ZnO/CsPbI_2_Br films show different peak shape in Figure 3b. Due to the multi‐sources of the oxygen element in the ligand containing ZnO film, we have differentiated peaks of O 1s into Zn—O, oxygen‐deficient (O_v_) and C=O components (representing CH_3_COO^−^) from the lowest to the highest binding energy, and the corresponding differentiated peaks were plotted in Figure 3c,d. Both intensities of C=O and O_v_ of the ZnO/CsPbI_2_Br film become weaker than those in pure ZnO film excluding Zn—O, indicating that partial content of CH_3_COO^−^ has been volatilized and more Zn atoms are bound to O atoms during the perovskite annealing process, which agrees well with the secondary volatilization to induce pinholes in perovskite films observed in SEM images. C=O moves toward a higher binding energy, indicating that the electron density around C=O is decreased.^[^ ^20^, ^46^, ^47^ ^]^ Therefore, the fact that Cs atoms and C=O move toward opposite directions in binding energy suggests that electrons of O in CH_3_COO^−^ can donate to Cs to form strong Cs‐Ac interaction to passivate traps of perovskite, which has been reported in previous publication.^[^ ^48^ ^]^ Whereas the peak positions of Pb 4f exhibit no shift between ZnO/CsPbI_2_Br and pure CsPbI_2_Br films (Figure S5a, Supporting Information), indicating that the ZnO film has negligible passivation effect to Pb atoms. Figure 2d also shows the existence of –NH_2_ and –OH groups in the ZnO film, whilst the –OH group cannot be recognized as the passivation units due to its electron withdrawing character, XPS spectrum does show weak interactions between –NH_2_ and perovskite (see Figure S5b, Supporting Information). However the XPS peak intensity of –COO– group is dozens of times stronger than that of –NH_2_ group, suggesting that the passivation effect of the trace amount of NH_2_ groups is a secondary factor. From the above information, we can conclude that CH_3_COO^−^ can form Cs–Ac interactions and effectively passivate traps of Cs atoms in inorganic CsPbI_2_Br perovskite (as schemed in Figure 1b), contributing to higher performance. Critically, excess organic ligands will create pinholes in perovskite films, only applying a 150 °C annealing of ZnO can we develop pinhole‐free and highly passivated perovskite films.

**Figure 4:**
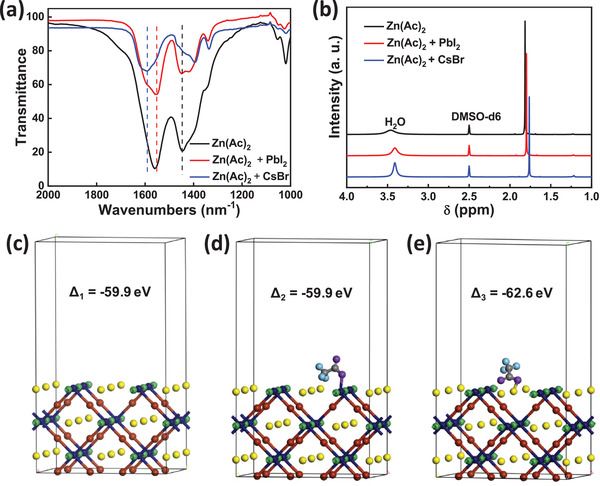
a) FTIR and b) ^1^H NMR of Zn(Ac)_2_, Zn(Ac)_2_ + PbI_2_, and Zn(Ac)_2_ + CsBr. The optimized structures of CsPbI_2_Br (110) surface with c) I vacancy, d) Pb—Ac bonding and e) Cs—Ac bonding. All atoms in (c–e) share the same colors with Figure 1.

To further verify the mechanism of interaction between acetate ion ligands in ZnO film and CsPbI_2_Br, we performed Fourier transform infrared (FTIR) spectra and ^1^H nuclear magnetic resonance (^1^H NMR) of pure Zn(Ac)_2_, Zn(Ac)_2_/PbI_2_, and Zn(Ac)_2_/CsBr mixtures. As shown in **Figure** **4**a, the peaks of Zn(Ac)_2_ in 1560 and 1445 cm^−1^ represent asymmetric and symmetric stretching of the carboxylate ion (−COO^−^). After adding CsBr into Zn(Ac)_2,_ the strong asymmetric stretching peak shifts to 1591 cm^−1^ and the symmetric stretching peak shifts to 1393 cm^−1^, indicating strong interactions between Ac^−^ and Cs atom. Whilst the minor shift of these peaks in the Zn(Ac)_2_/PbI_2_ mixture suggests negligible interactions between Ac^−^ and Pb atom.^[^ ^48^ ^]^ The result is also confirmed by ^1^H NMR. In Figure 4b, all samples are calibrated by the peak of dimethyl sulfoxide‐d6 (DMSO‐d6) locating at 2.50 ppm, with peaks in 3.30–3.50 ppm are contributed by water. After mixing PbI_2_ and CsBr with Zn(Ac)_2_, the original peak of Ac^−^ from Zn(Ac)_2_ shifts from 1.81 to 1.80 and 1.76 ppm respectively, indicating that Ac^−^ is more likely to interact with Cs rather than Pb atoms.^[^ ^49^ ^]^ We continued to calculate the formation energy of perovskite film with and without the presence of Ac^−^ bonding by the density functional theory (DFT) method, with detailed calculation information presented in the Supporting Information. In Figure 4c, the formation energy of I vacancy in CsPbI_2_Br is −59.9 eV, which retains after bonding Ac^−^ with the Pb atom (Figure 4d), but turns more negative to −62.6 eV with the formation of Cs—Ac bonding (Figure 4e). This suggests that the Cs—Ac bonding can help to form more stable CsPbI_2_Br structure than the I vacancy and Pb—Ac bonding, which is consistent with previous report that the Ac^−^ can strongly stabilize CsPbI_2_Br through Cs‐Ac interactions.^[^ ^48^ ^]^ The comprehensive analysis of XPS, FTIR, ^1^H NMR and theoretical calculation therefore confirm that Ac^−^ is more easily to interact with Cs atoms rather than Pb atoms, which will effectively passivate traps of Cs atoms in inorganic CsPbI_2_Br perovskite to impact on device performance.

**Figure 5:**
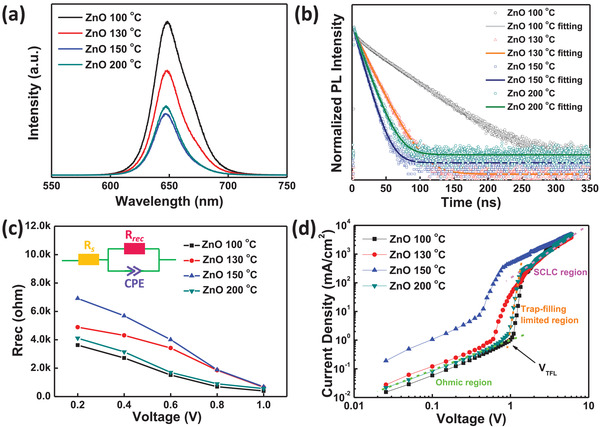
a) Steady‐state and b) time‐resolved photoluminescence spectra of inorganic CsPbI_2_Br deposited on different temperature annealing ZnO. c) Rrec obtained from fitting the Nyquist plots through equivalent circuit in the inset, for PSCs incorporating temperature annealing ZnO. d) Dark current–voltage curves from electron‐only devices with the structure of ITO/ZnO/CsPbI_2_Br/ PC_61_BM/Ag.

We proceeded to probe charge recombination dynamics by using steady‐state photoluminescence (PL). **Figure** **5**a shows that the PL spectra of ZnO/CsPbI_2_Br films, and the 150 °C annealed ZnO based film shows the strongest PL quenching, meaning the most effective electron extraction from perovskite to ZnO. In order to quantize this process, time‐resolved photoluminescence (TRPL) was employed to record carrier lifetime of perovskite films. Figure 5b illustrates the TRPL curves of ZnO/CsPbI_2_Br films, with elevated annealing temperature of ZnO from 100 to 130, 150 and 200 °C, corresponding lifetime firstly decreases from 41.9 to 15.0 and 8.9 ns then slightly increases to 9.2 ns, again confirming that the 150 °C annealed ZnO based perovskite film have the fastest electron transport ability and least recombination.^[^ ^50^ ^]^

Impedance spectroscopy measurements were utilized to investigate bulk and interface charge transport properties. Figure S6a–d (Supporting Information) illustrate the impedance plots of ZnO‐based (100, 130, 150, and 200 °C annealing) devices under 1 sun illumination at a series of applied voltage, and the bias loading mode is recorded in Figure S6f (Supporting Information). All impedance plots can be well fitted by the equivalent circuit inserted in Figure 5c, and relevant fitting parameters *R*_rec_, *R*_s_, and CPE represent recombination resistance, series resistance and constant phase element respectively.^[^ ^51^ ^]^ Figure 5c shows the decreased *R*_rec_ of all devices along the increased applied voltage, and 150 °C annealed ZnO based device has the largest *R*_rec_ than all other devices, which indicates the maximally declined recombination in 150 °C annealed ZnO based device, in accordance with the electron transport and efficiency results. This result could be also related to the reduced trap density at the 150 °C annealed ZnO/CsPbI_2_Br interface compared to more recombination centers in 100, 130, and 200 °C annealed ZnO based perovskite films. Thus we fabricated electron‐only devices with the structure of ITO/ZnO/CsPbI_2_Br/PC_61_BM/Ag to quantitatively compute the trap density. In Figure 5d, *J*–*V* curves of double logarithmic coordinates can be divided into three parts, Ohmic, trap‐filling limited and space charge limited current (SCLC) regions, and the transition point between Ohmic and trap‐filling limited regions represents the trap‐filled limit voltage (*V*_TFL_), from which we can calculate trap density (*n*_t_) from *V*_TFL_ = *en*_t_ *L* ^2^/2*εε*_0_, where *ε*_0_ and *ε* are the vacuum permittivity and the relative dielectric constant of CsPbI_2_Br respectively, *L* is the thickness of the CsPbI_2_Br film.^[^ ^52^ ^]^ The calculated trap density of 100, 130, 150, and 200 °C annealed ZnO based device is 3.46 × 10^16^, 1.57 × 10^16^, 9.44 × 10^15^, and 2.89 × 10^16^ cm^−3^ respectively. The reduced trap density of 150 °C annealed ZnO based device could be attributed to the effective interfacial passivation of the CsPbI_2_Br film by the CH_3_COO^−^ ligands in ZnO film.

**Figure 6:**
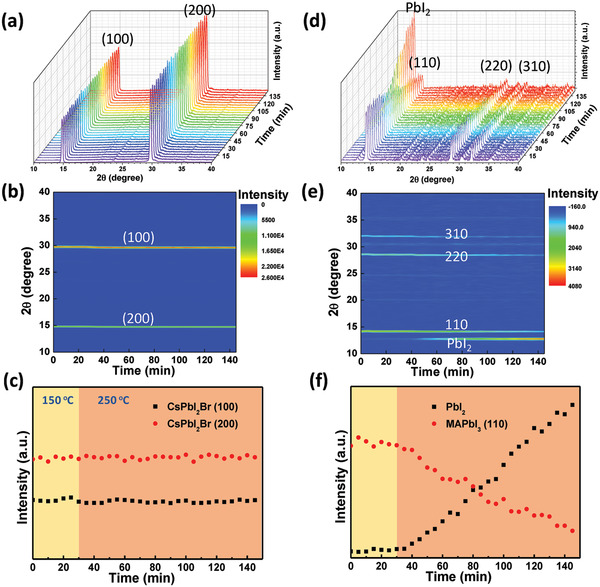
In‐situ high temperature XRD and their corresponding mapping figures and evolution of characteristic diffraction peak of a–c) CsPbI_2_Br and d–f) MAPbI_3_ films. The annealing temperature of perovskite during XRD testing were 150 °C for 30 min first then increased to 250 °C for another 120 min.

We continued to probe the thermal stability by recording in‐situ XRD at an annealing temperature of 150 °C first for 30 min and then increased to 250 °C for another 120 min. **Figure** **6**a,b illustrates the XRD original data of CsPbI_2_Br film and its mapping figure, and the characteristic diffraction peaks of cubic structure CsPbI_2_Br at 14.8° and 29.7° represent to the (100) and (200) crystal planes respectively.^[^ ^33^, ^53^ ^]^ Figure 6c plots the peak values of CsPbI_2_Br (100) and (200) planes, from which we can find that the CsPbI_2_Br film exhibit almost identical intensity of various diffraction peaks, even elevated to a temperature of 250 °C, indicating the excellent thermal stability of CsPbI_2_Br. Then we also measured in‐situ high temperature XRD of organic‐inorganic hybrid perovskite MAPbI_3_ under the same condition. Figure 6d,e shows the XRD original data of MAPbI_3_ and its corresponding mapping figure, and the characteristic diffraction peaks of MAPbI_3_ at 14.3°, 28.5°, and 32.1° represent to the (110), (220), and (310) crystal planes respectively, and the diffraction peak at 12.8° can be attributed to PbI_2_.^[^ ^51^ ^]^ We can find that all diffraction peaks of MAPbI_3_ became weaker and the peak of PbI_2_ got stronger during the heating process. For a clearer comparison, the strongest peak of MAPbI_3_ (110) and the peak of PbI_2_ are recorded in Figure 6f. During the first 30 min annealing under 150^ ^°C, there are not obvious change of diffraction peaks, but the intensity of PbI_2_ increases and MAPbI_3_ (110) decreases continuously when the annealing temperature was elevated to 250^ ^°C. Therefore the organic‐inorganic hybrid perovskite is less thermally stable than the inorganic CsPbI_2_Br. Afterward we fabricate full devices of MAPbI_3_ and CsPbI_2_Br to track the thermal stability and performance evolution of MAPbI_3_ and CsPbI_2_Br PSCs under 85 °C in N_2_ glovebox (Figure S7, Supporting Information). MAPbI_3_‐based device is almost dead after 6 h and CsPbI_2_Br‐based device can maintain 75% of the initial performance over 20 h, consistent with the in‐situ high temperature XRD, and the quick decreased performance may be associated with the destruction of interface between polyTPD and silver electrode.

**Figure 7:**
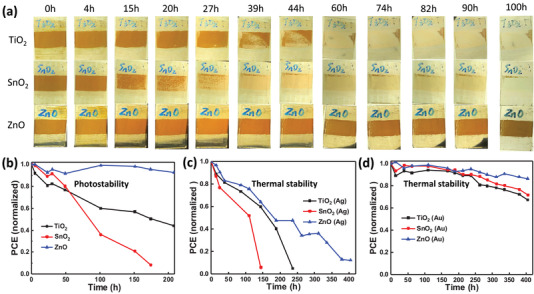
a) Digital photos showing the degradation of inorganic CsPbI_2_Br films upon 100 mW cm^−2^ LED white light illumination under ≈10% relative humidity. b) Normalized performance of encapsulated CsPbI_2_Br PSCs with different ETMs under 100 mW cm^−2^ LED white light illumination in ambient atmosphere. Normalized performance of CsPbI_2_Br PSCs with c) Ag or d) Au electrodes using different ETMs under 85 °C in N_2_ glovebox.

We subsequently applied continuous 100 mW cm^−2^ white light LED on ETMs/CsPbI_2_Br films under ≈10% RH in air to observe the film degradation. As shown in **Figure** **7**a, the CsPbI_2_Br film on SnO_2_ began to decompose apparently after 15 h and nearly disappeared after 39 h. The TiO_2_/CsPbI_2_Br film became transparent after 60 h and the ZnO/CsPbI_2_Br film showed little decomposition after 100 h. This result demonstrates the superior photostability of the ZnO/CsPbI_2_Br film, consistent with the suppression function of ZnO film in photoinduced halide phase separation of CsPbI_2_Br. We also tracked the performance evolution of encapsulated CsPbI_2_Br devices under continuous 100 mW cm^−2^ white LED illumination (Figure 7b). The 150 °C annealed ZnO based device kept 90% of the initial performance after 200 h illumination, much higher than the TiO_2_‐ and SnO_2_‐ based devices (below 50%). Finally, we tracked the thermal stability of CsPbI_2_Br PSCs using different ETMs under 85 °C in N_2_ glovebox. Similar to the aforementioned film thermal stability, all devices using Ag electrode exhibit quick performance reduction, although the ZnO based CsPbI_2_Br PSC show the best thermal stability (Figure 7c). However, the overall thermal stability of CsPbI_2_Br PSCs can be enhanced through the replacement of Ag electrode with Au electrode to inhibit the reaction of Ag with perovskite, which has been well reported in the literature.^[^ ^54^ ^]^ As shown in Figure 7d, with Au as the top electrode, the 150 °C annealed ZnO based device maintained 86% of the initial performance after 400 h continuous thermal annealing under 85 °C, much higher than the TiO_2_‐ (67%) and SnO_2_‐ (71%) based devices.

## 3 Conclusions

We have fabricated all‐layer‐doping‐free planar heterojunction inorganic CsPbI_2_Br PSCs using organic ligands (acetate ions) armored ZnO as ETM, acquiring an encouraging photovoltaic performance of 16.84%, which is among the highest PCE of inorganic CsPbI_2_Br PSCs. Meantime, ZnO can remarkably suppress the photoinduced phase separation of CsPbI_2_Br film compared to SnO_2_, with the ZnO/CsPbI_2_Br film can maintain integrity in ≈10% RH over 100 h and the encapsulated ZnO‐based PSCs can maintain 90% PCE over 200 h, upon continuous 100 mW cm^−2^ white light LED illumination. The ZnO‐based PSCs can maintain 86% PCE over 400 h continuous thermal annealing under 85 °C. The enhanced performance, photostability and thermal stability arise from the proper amount of acetate ions residue in the ZnO film by optimizing the thermal annealing temperature, which can not only maintain excellent crystallinity of CsPbI_2_Br but also effectively passivate perovskite through the formation of cesium/acetate interactions that are confirmed through the comprehensive analysis of XPS, FTIR, ^1^H NMR, and theoretical calculation. Our work provides a simple and convenient method to fabricate high efficiency and stable inorganic CsPbI_2_Br PSCs.

## 4 Experimental Section

##### Materials

Detailed synthesis information of methylammonium iodide (MAI) and titanium dioxide (TiO_2_) can be found in the previous work.^[^ ^52^ ^]^ Lead (II) iodide (PbI_2_, 99.99%) and polyTPD was purchased from Xi'an Polymer Light Technology in China. Cesium bromide (CsBr, 99.99%), *N*,*N*‐Dimethylformamide (DMF, anhydrous, 99.8%), Dimethyl sulfoxide (DMSO, anhydrous, 99.9%) and were purchased from Sigma‐Aldrich. Zinc acetate (Zn(CH_3_COO)_2_·2H_2_O, 99.99%) was purchased from Aladdin. The tin(IV) dioxide (SnO_2_, 15% in H_2_O) was obtained from Alfa Aesar. High transmittance ITO (resistance ≈15 Ω sq^−1^, maximum transmittance ≈94% at ≈550 nm, size of 20 × 15 × 0.7 mm^3^) was purchased from You Xuan Ltd.

##### Device Fabrication

Unless otherwise stated, all of fabrication were according to the previous work^[^ ^35^, ^55^ ^]^ SnO_2_ solution was prepared by mixing SnO_2_ precursor (Alfa Aesar) and deionized water at a ratio of 1:6, and was stirred at least 2 h before use, then was spin‐coated on ITO whereafter annealed at 150 °C for 30 min to form a compact SnO_2_ layer. ZnO precursor was prepared by dissolving 200 mg Zinc acetate and 56 µL ethanolamine in 2 mL 2‐methoxyethanol, and was stirred at least 12 h before use, then was spin‐coated on ITO and annealed at several temperature (100, 130, 150, and 200 °C) for 30 min to obtain ZnO layer (40 nm) with different amount of organic ligands residue. 1 m PbI_2_:CsBr (1:1) dissolving in DMF:DMSO (1:9) was deposited on metal oxide at 1500 rpm 10 s then 4000 rpm 30 s, then was blown by fan for 60 s, finally was annealed at 50 °C for 30 s and 240^ ^°C for 60 s successively to form CsPbI_2_Br film.

##### Characterizations

Unless otherwise stated, all of characterization methods and techniques can be found in the previous work.^[^ ^35^, ^51^ ^]^ Briefly, the device *J*–*V* characterization was performed under AM 1.5G (100 mW cm^−2^) using a Newport 3A solar simulator in air at room temperature. The light intensity was calibrated using a standard silicon reference cell certified by the National Renewable Energy Laboratory (NREL, USA). *J*–*V* characteristics were recorded using *J*–*V* sweep software developed by Ossila Ltd. (UK) and a Keithley (USA) 2612B source meter unit. An aperture mask was placed over the devices to accurately define a test area of 2.12 mm^2^ on each pixel and to eliminate the influence of stray and wave guided light. The *J*–*V* scan rate was 50 mV s^−1^. External quantum efficiency (EQE) was measured with a Zolix (China) EQE system equipped with a standard Si diode. The steady‐state PL (Flex One, Zolix, China) and time‐resolved PL (PicoQuant, Fluo Time 300, Germany) spectra were obtained utilizing 532 and 520 nm CW laser respectively. The in‐situ high temperature XRD data were obtained using X‐ray diffractometer (D8 DISCOVER, China). X‐ray photoelectron spectra were acquired by X‐ray photoelectron spectrometer (ESCALAB 250Xi, America). Infrared absorption was measured using Fourier transform infrared spectroscopy (spectrum two, PerkinElmer, America), mixtures of Zn(Ac)_2_/PbI_2_ and Zn(Ac)_2_/CsBr were obtained by dissolving in DMSO then drying in vacuum oven at about 100 °C. NMR spectra were measured in DMSO‐d6 solvent using a BRUKER DRX500 spectrometer with tetramethylsilane (TMS) as the internal standard.

## Conflict of Interest

The authors declare no conflict of interest.

## Supporting information

Supporting Information
