# Supplementary material for: Organic Ligands Armored ZnO Enhances Efficiency and Stability of CsPbI2Br Perovskite Solar Cells
Source: Adv Sci (Weinh). 2020 Sep 27;7(21):2000421. doi: 10.1002/advs.202000421 (PMC7610334; doi:10.1002/advs.202000421)
Supplement: Supplementary file 1 — Supporting Information [file ADVS-7-2000421-s001.pdf]

© 2020 Wiley-VCH GmbH

ADVANCED SCIENCE
Open Access

Supporting Information

for *Adv. Sci.*, DOI: 10.1002/advs.202000421

Organic Ligands Armored ZnO Enhances Efficiency and Stability of CsPbI2Br Perovskite Solar Cells

*Pang Wang, Hui Wang, Yuchao Mao, Huijun Zhang, Fanghao Ye, Dan Liu and Tao Wang**

Supporting Information

Organic Ligands Armored ZnO Enhances Efficiency and Stability of $CsPbI_2Br$ Perovskite Solar Cells

*Pang Wang, Hui Wang, Yuchao Mao, Huijun Zhang, Fanghao Ye, Dan Liu and Tao Wang**

P. Wang, H. Wang, Y. Mao, H. Zhang, F. Ye, Dr. D. Liu, Prof. T. Wang

School of Materials Science and Engineering, Wuhan University of Technology, Wuhan, 430070, China

*E-mail: twang@whut.edu.cn

P. Wang, H. Wang, Y. Mao, H. Zhang, F. Ye, Dr. D. Liu, Prof. T. Wang

State Key Laboratory of Silicate Materials for Architectures, Wuhan University of Technology, Wuhan, 430070, China

DFT calculating methods:

The geometric optimization and total energy calculations were performed in Materials Studio software on the basis of DFT. Gradient-corrected functionals GGA and Standard Perdew-Burke-Ernzerhof (PBE) functionals were used to optimize the geometric structure of the $CsPbI_2Br$ crystals, PbIBr, CsI, PbBrAc, PbBr and CsAc. The multipolar expansion with all electron core treatment was adopted to be hexadecapole. A global orbital cutoff scheme was applied for the single-point energy calculation. The energy convergence tolerance was less than 1.0×10^{-5} Ha per atom. The atomic positions were considered to be fully relaxed until the max-displacement converged under 0.005 Å with residual forces below 0.002 Ha $Å^{-1}$. The surface formation energy of the functional $CsPbI_2Br$ slab with I Vacancy was calculated by: $\Delta_1 = 12*E_{CsPbI2Br} - 12*E_{CsI} - 11E_{PbBrI} - E_{PbBr}$. The surface formation energy of the functional $CsPbI_2Br$ slab with I Vacancy (Pb-Ac bonding) was calculated by: $\Delta_2 = 12*E_{CsPbI2Br} - 12*E_{CsI} - 11E_{PbBrI} - E_{PbBrAc}$. The surface formation energy of the functional $CsPbI_2Br$ slab with I Vacancy (Cs-Ac bonding) was calculated by: $\Delta_3 = 12*E_{CsPbI2Br} - 11*E_{CsI} - 12E_{PbBrI} - E_{CsAc}$, where $E_{CsPbI2Br}$, E_{CsI}, E_{PbBrI}, E_{PbBr}, E_{PbBrAc} and

E_{CsAc} are respectively the bulk energy of $CsPbI_2Br$ crystals, CsI, PbIBr, PbBr, PbBrAc and CsAc.

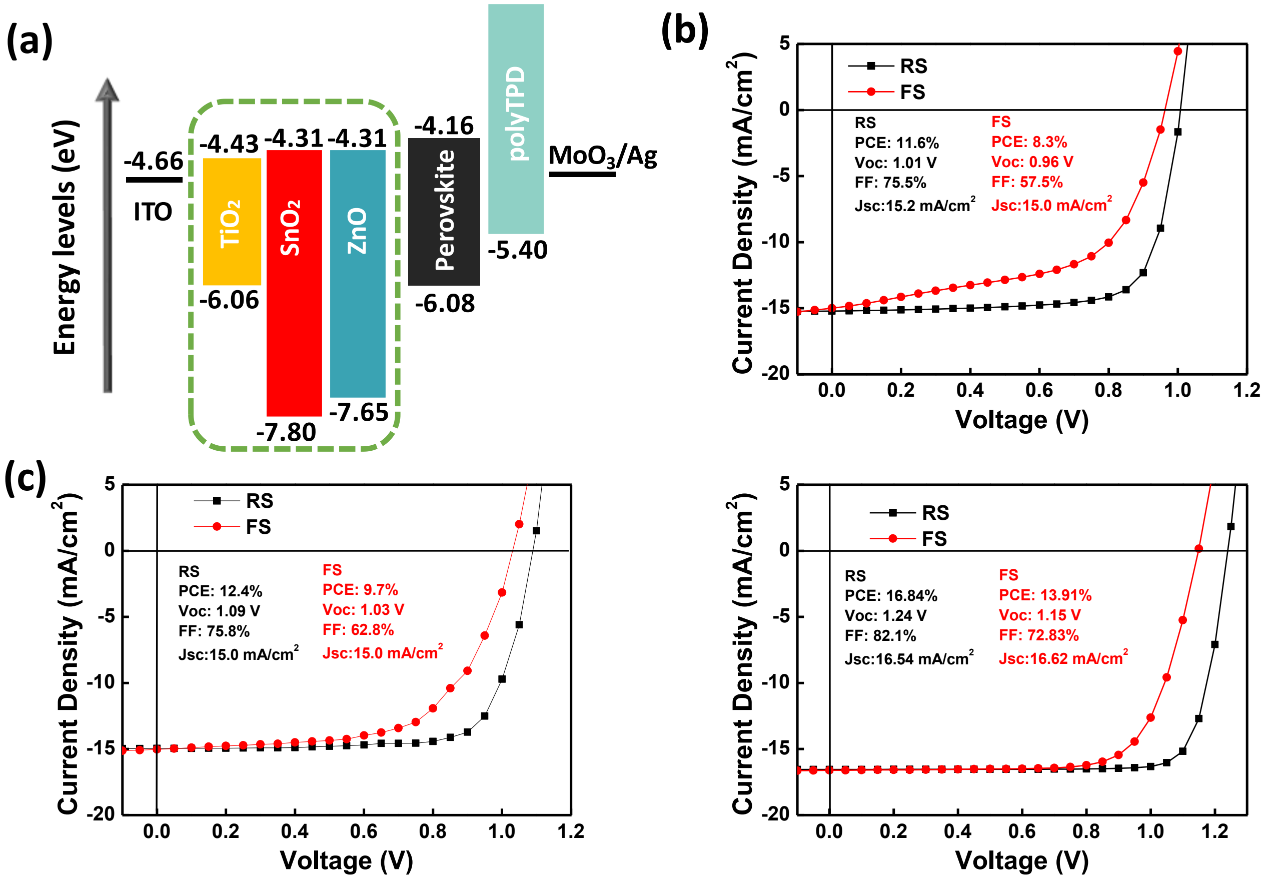

Figure S1. (a) Energy level alignments of PSCs. The best J-V curves of all-inorganic $CsPbI_2Br$ PSCs from forward and reverse scans using (b) TiO_2, (c) SnO_2 and (d) ZnO as ETMs. The energy levels of ZnO correspond to the one upon thermal annealing at 150 ^{o}C.

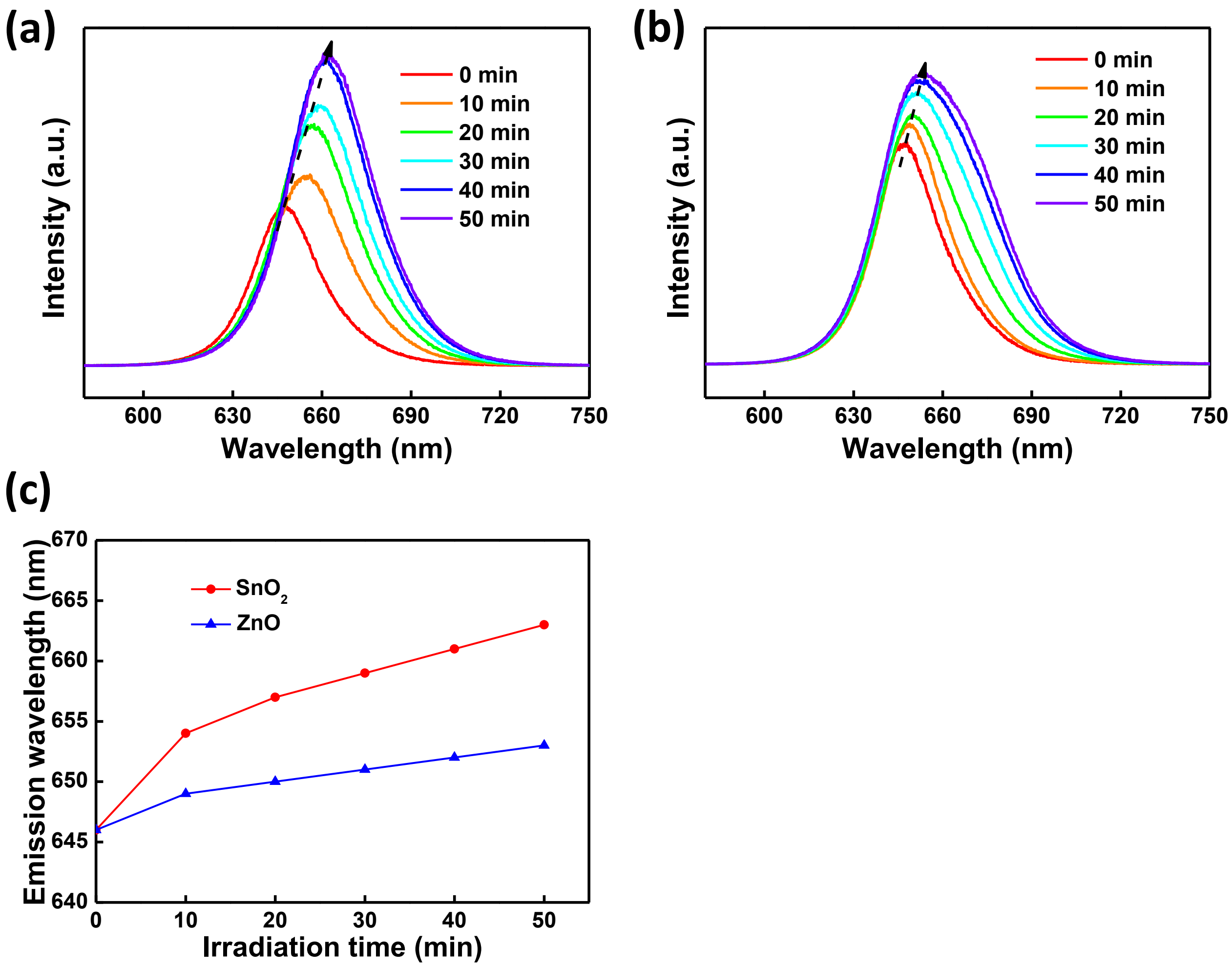

Figure S2. Steady-state photoluminescence spectra of (a) $SnO_2/CsPbI_2Br$ and (b) $ZnO/CsPbI_2Br$ upon continuous 1 sun illumination under 20-30% RH in ambient atmosphere. (c) Evolution of corresponding emission wavelength of $SnO_2/CsPbI_2Br$ and $ZnO/CsPbI_2Br$ films.

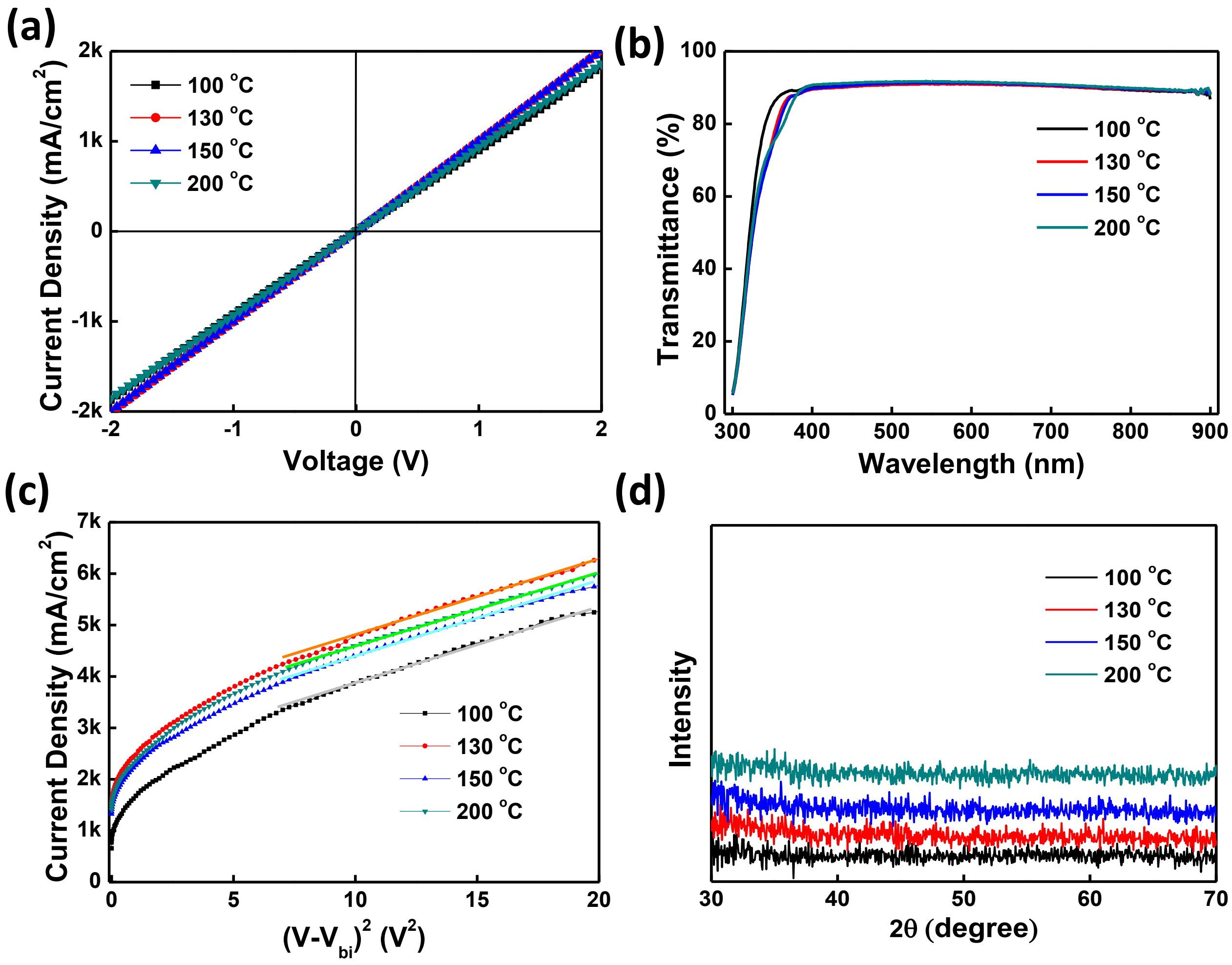

Figure S3. (a) Conductivity, (b) transmittance, (c) mobility and (d) XRD of different temperature annealed ZnO.

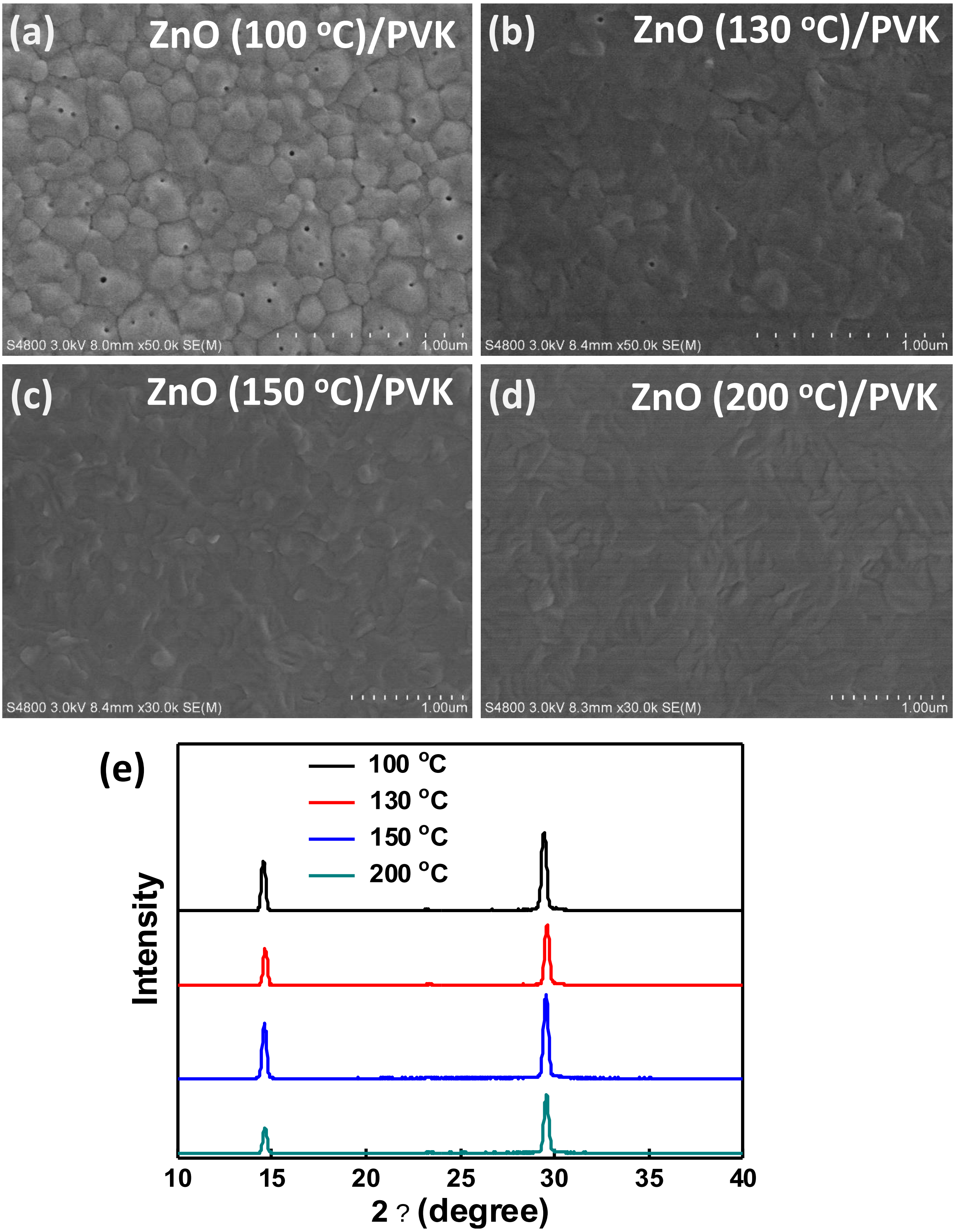

Figure S4. SEM figures of $CsPbI_2Br$ films cast on ZnO annealed at different temperatures: (a) 100 °C, (b) 130 °C, (c) 150 °C, and (d) 200 °C. **(e)** XRD of $CsPbI_2Br$ films cast on ZnO annealed at different temperatures.

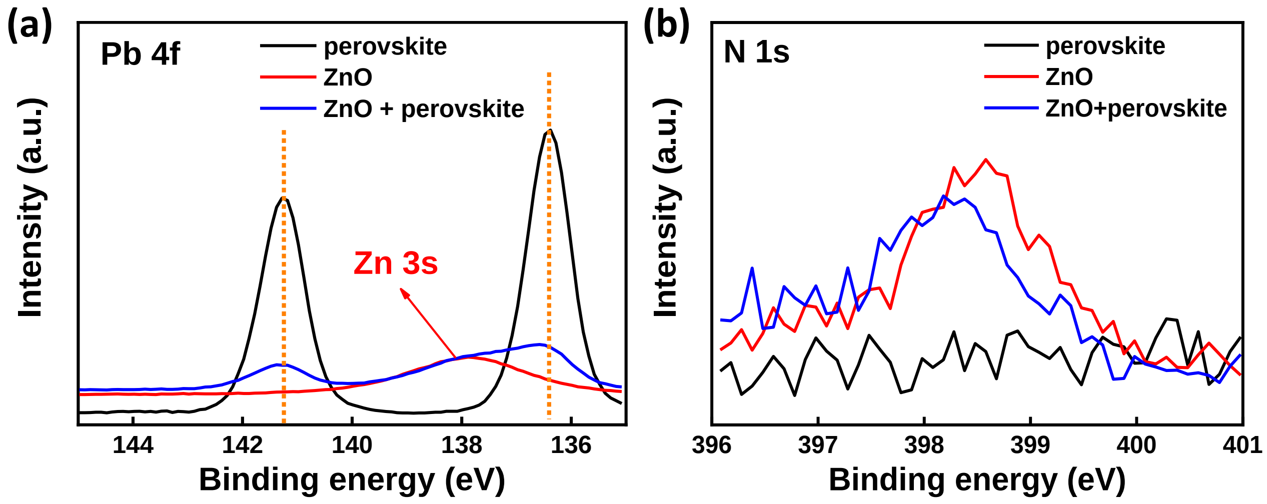

Figure S5. XPS spectra of (a) Pb 4f and (b) N 1s in $CsPbI_2Br$, ZnO and ZnO/$CsPbI_2Br$ films.

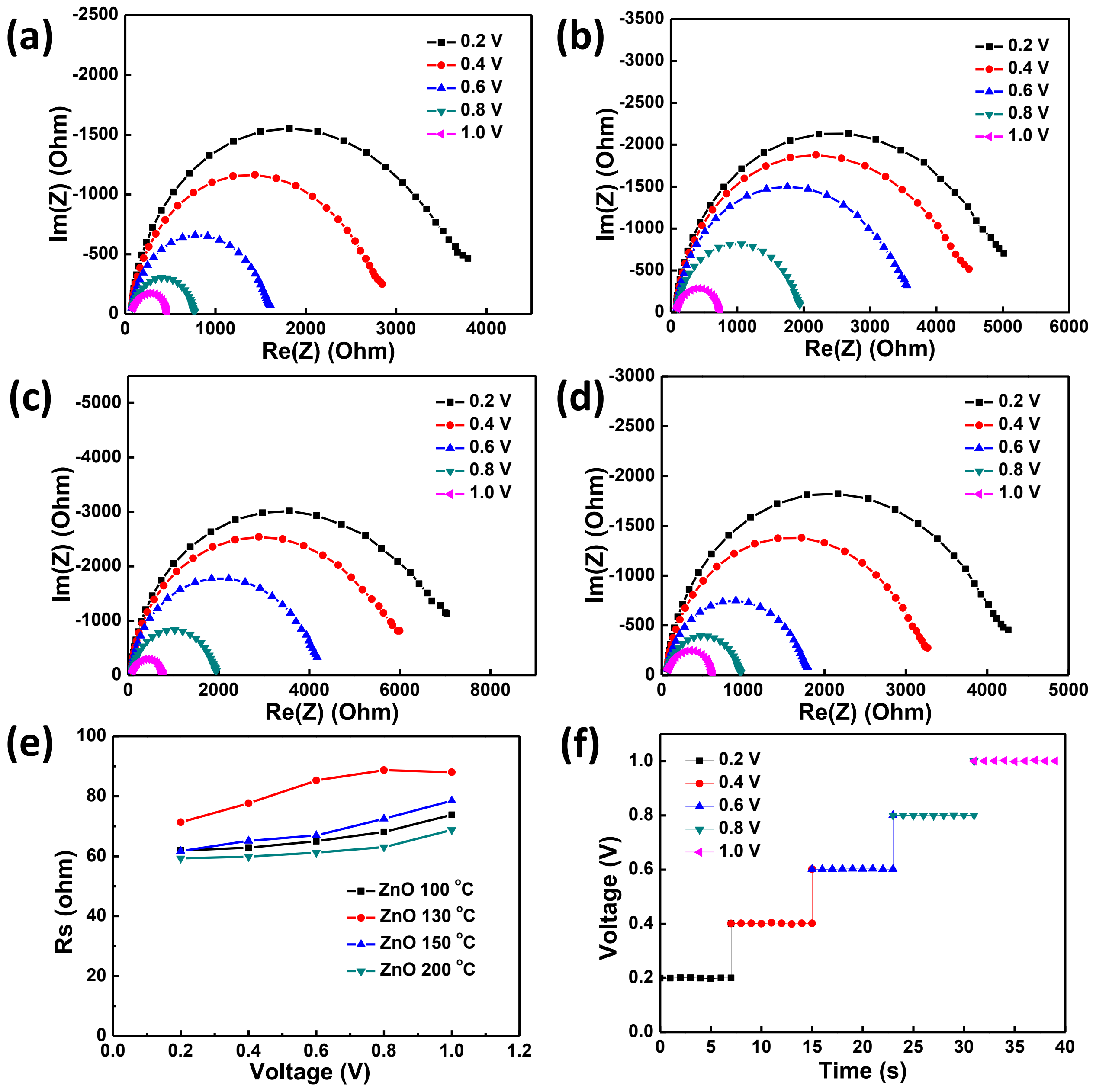

Figure S6. Impedance plots measured under 1 sun illumination from devices using different temperature annealed ZnO: (a) 100 °C, (b) 130 °C, (c) 150 °C and (d) 200 °C. (e) R_s of corresponding devices obtained from fitting the Nyquist plots. (f) Voltage variations during the whole testing process.

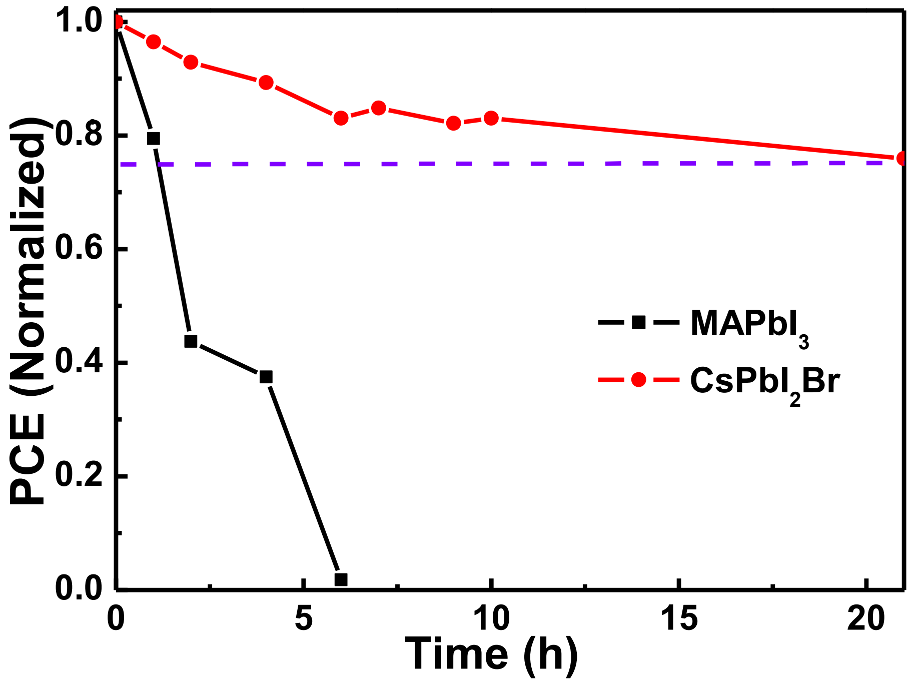

Figure S7. Normalized performance of encapsulated $MAPbI_3$ and $CsPbI_2Br$ PSCs under 85 ^{o}C in N_2 golvebox.

Table S1. Biexponential decay fitting parameters of TRPL for $CsPbI_2Br$ films cast on different temperature annealed ZnO.

**Temperature (^{o}C)**	**τ_1 (ns)**	**τ_2 (ns)**	**t (ns)**
100	6.2	43.2	41.9
130	13.3	19.2	15.0
150	7.9	12.3	8.9
200	9.2	13.4	11.2

Biexponential fitting function: $i = A_1 \exp(-t/\tau_1) + A_2 \exp(-t/\tau_2)$

Table S2. A summary of reported maximum PCEs of inorganic perovskite solar cells based on $CsPbI_2Br$.

**ETLs**	**Fabrication**	**Device structure**	**PCE (%)**	**Ref**
p-i-n				
PCBM		ITO/PEDOT:PSS/$CsPbI_2Br$/PCBM/BCP/Al	6.8	[1]
c-Nb_2O_5/	Bilayer	ITO/NiOx/$CsPbI_2Br$/c-Nb_2O_5/PCBM/Ag	11.74	[2]

PCBM				
ZnO@ C60	Bulk doping & bilayer	FTO/NiOx/$CsPbI_2Br$/ZnO@C60/Ag	13.3	[3]
PCBM/ C60	Bulk doping	ITO/P3CT/ $CsPb_{0.98}Ni_{0.02}I_2Br$/PCBM/C60/ BCP/Ag	13.88	[4]
TiO_2	HTM doping	FTO/NiMgLiO/$CsPbI_2Br$/TiO_2/Ag	14.0	[5]
Nb_2O_5		ITO/NiOx/$CsPbI_2Br$/Nb_2O_5/Ag	14.45	[6]
C-TiO_2	HTM doping	FTO/NiMgLiO/$CsPbI_2Br$/C-TiO_2/Sb	14.8	[7]
n-i-p				
Ca/C60	Bilayer	ITO/Ca/C60 /$CsPbI_2Br$/TAPC/TAPC: MoO_3/Ag	11.8	[8]
Ca/C60	Bilayer	ITO/Ca/C60/$CsPbI_2Br$/TAPC/TAPC:MoO_3/ MoO_3/Ag	13.0	[9]
TiO_2	HTM doping	FTO/TiO_2/$CsPbI_2Br$/Spiro-MeOTAD/Ag	9.8	[10]
TiO_2	HTM doping	FTO/TiO_2/$CsPbI_2Br$/Spiro-MeOTAD/Ag	10.6	[11]
TiO_2	HTM doping	FTO/TiO_2/$CsPbI_2Br$/Spiro-MeOTAD/Au	10.7	[12]
TiO_2	HTM doping	ITO/TiO_2/$CsPbI_2Br$/P3HT/Au	12.0	[13]
TiO_2	Bulk & HTM doping	FTO/TiO_2/ $CsPb_{0.9}Zn_{0.1}I_2Br$/Spiro-OMeTAD/Au	13.6	[14]
TiO_2	HTM doping & bilayer	FTO/TiO_2/$CsPbI_2Br$/$CsPbI_3$ QDs/PTAA/Au	14.4	[15]
TiO_2	HTM doping & bilayer	FTO/TiO_2/$CsPbI_2Br$/$CsPbI_2Br$ QDs/PTAA/Au	14.8	[16]
TiO_2	Bulk & HTM doping	FTO/TiO_2/$CsPbI_2Br(Eu(Ac)_3)$/Spiro-OMeTAD/Au	15.25	[17]
TiO_2	Bulk & HTM doping	FTO/TiO_2/ $CsPbI_{2-x}Br(Ac)_x$/Spiro-OMeTAD/Au	15.56	[18]
c-TiO_2	HTM doping	ITO/c-TiO_2/$CsPbI_2Br$/Spiro-MeOTAD/Au	**16.07**	[19]
TiO_2	HTM doping	FTO/TiO_2/$CsPbI_2Br$/$CuBr_2$/Spiro-OMeTAD/MoO_3/Ag	**16.15**	[20]
c-TiO_2/ mp-TiO_2	Bulk doping & bilayer	FTO/c-TiO_2/mp-TiO_2/$CsPb_{0.98}Sr_{0.02}I_2Br$/P3HT/Au	11.2	[21]
c-TiO_2/ mp-TiO_2	HTM doping & bilayer	FTO/c-TiO_2/mp-TiO_2/$CsPbI_2Br$/Bi_2Te_3/Spiro-OMeTA D/Ag	11.96	[22]
c-TiO_2/ mp-TiO_2	Bulk and HTM doping & bilayer	FTO/c-TiO_2/mp-TiO_2/$CsPb_{0.95}Eu_{0.05}I_2Br$/Spiro-MeOT AD/Au	13.7	[23]
c-TiO_2/ mp-TiO_2	Bulk and HTM doping & bilayer	FTO/c-TiO_2/mp-TiO_2/$CsPb_{0.8}Ba_{0.2}I_2Br$/Spiro-OMeTA D/Au	14.0	[24]
c-TiO_2/ mp-TiO_2	Bulk doping & Bilayer	FTO/c-TiO_2/mp-TiO_2/ BaI_2:$CsPbI_2Br$/P3HT/Au	14.85	[25]
SnO_2	Bulk doping	ITO/SnO_2/$CsPbI_2Br$:Nb/Carbon	10.42	[26]
SnO_2	Bulk doping	ITO/SnO_2/$CsPbI_2Br$:$Pb(SCN)_2$/P3HT/Ag	12.22	[27]
SnO_2	Bulk doping	ITO/SnO_2/$CsPb_{0.97}Y_{0.03}I_2Br$/P3HT/Au.	13.25	[28]
SnO_2	HTM doping	ITO/SnO_2/$CsPbI_2Br$/Spiro-OMeTAD/MoO_3/Ag	13.5	[29]
SnO_2		ITO/SnO_2/$CsPbI_2Br$/P3HT/Au	13.91	[30]
SnO_2	Mixed HTM	ITO/SnO_2/$CsPbI_2Br$/PBDTTT-E-T:IEICO/MoO_3/Ag	14.03	[31]
SnO_2	HTM doping & bilayer	ITO/SnO_2/$CsPbI_2Br$/BrBeAI-2/Spiro-OMeTAD/Au	14.63	[32]

SnO_2	HTM doping	ITO/SnO_2/$CsPbI_2Br$/Spiro-OMeTAD/Au	15.17	[33]
SnO_2	HTM doping & bilayer	ITO/SnO_2/$CsPbI_2Br$/$(CsPbI_2Br)_{1-x}(CsPbI_3)_x$/ Spiro-MeOTAD/Au	15.50	[34]
SnO_2	HTM doping & bilayer	ITO/SnO_2/$CsPbI_2Br$/CsBr/Spiro-OMeTAD/Au	**16.37**	[35]
SnO_2	HTM doping	ITO/SnO_2/$CsPbI_2Br$/PTAA/Au	**16.58**	[36]
SnO_2/ PN4N	Bilayer	ITO/SnO_2/PN4N/$CsPbI_2Br$/PDCBT/MoO_3/Ag	**16.2**	[37]
ZnO@ SnO_2	HTM doping	FTO/ZnO@SnO_2/$CsPbI_2Br$/Sprio-OMeTAD/MoO_3/Ag	14.35	[38]
SnO_2/ ZnO	HTM doping & bilayer	ITO/SnO_2/ZnO/$CsPbI_2Br$/Spiro-OMeTAD/MoO_3/Ag	14.6	[39]
ZnO/ SnO_2	bilayer	ITO/ZnO/SnO_2/$CsPbI_2Br$/PSQ2/MoO_3/Ag	15.5	[40]
**ZnO**	**w/o doping & single layer**	**ITO/ZnO/$CsPbI_2Br$/polyTPD/MoO_3/Ag**	**16.84**	**This work**

References:

[1] R. E. Beal, D. J. Slotcavage, T. Leijtens, A. R. Bowring, R. A. Belisle, W. H. Nguyen, G. F. Burkhard, E. T. Hoke, M. D. McGehee, *J. Phys. Chem. Lett.* **2016**, *7*, 746.

[2] D. Han, Y. Xin, Q. Yuan, Q. Yang, Y. Wang, Y. Yang, S. Yi, D. Zhou, L. Feng, Y. Wang, *Sol. RRL* **2019**, *3,* 1900091.

[3] C. Liu, W. Li, C. Zhang, Y. Ma, J. Fan, Y. Mai, *J. Am. Chem. Soc.* **2018**, *140*, 3825.

[4] L. Chen, L. Wan, X. Li, W. Zhang, S. Fu, Y. Wang, S. Li, H. Wang, W. Song, J. Fang, *Chem. Mater.* **2019**, *31*, 9032.

[5] S. Zhang, W. Chen, S. Wu, R. Chen, Y. Huang, Z. Yang, J. Li, L. Han, W. Chen, *J. Mater. Chem. A* **2019**, *7*, 18603.

[6] X. Liu, Y. Xiao, Q. Zeng, J. Jiang, Y. Li, *J. Phys. Chem. Lett.* **2019**, *10*, 6382.

[7] S. Zhang, W. Chen, S. Wu, R. Chen, Z. Liu, Y. Huang, Z. Yang, H. Zhu, J. Li, L. Han, W. Chen, *ACS Appl. Mater. Interfaces* **2019**, *11*, 43303.

[8] C. Y. Chen, H. Y. Lin, K. M. Chiang, W. L. Tsai, Y. C. Huang, C. S. Tsao, H. W. Lin, *Adv. Mater.* **2017**, *29*, 1.

[9] H. Lin, C. Chen, B. Hsu, Y. Cheng, W. Tsai, Y. Huang, C. Tsao, H. Lin, *Adv. Funct. Mater.* **2019**, *29*, 1905163.

[10] R. J. Sutton, G. E. Eperon, L. Miranda, E. S. Parrott, B. A. Kamino, J. B. Patel, M. T. Hörantner, M. B. Johnston, A. A. Haghighirad, D. T. Moore, H. J. Snaith, *Adv. Energy Mater.* **2016**, *6*, 1502458.

[11] Y. Wang, T. Zhang, F. Xu, Y. Li, Y. Zhao, *Sol. RRL* **2018**, *2*, 1700180.

[12] J. K. Nam, S. U. Chai, W. Cha, Y. J. Choi, W. Kim, M. S. Jung, J. Kwon, D. Kim, J. H. Park,

Nano Lett. **2017**, *17*, 2028.

[13] Q. Zeng, X. Zhang, X. Feng, S. Lu, Z. Chen, X. Yong, S. A. T. Redfern, H. Wei, H. Wang, H. Shen, W. Zhang, W. Zheng, H. Zhang, J. S. Tse, B. Yang, *Adv. Mater.* **2018**, *30*, 1.

[14] H. Sun, J. Zhang, X. Gan, L. Yu, H. Yuan, M. Shang, C. Lu, D. Hou, Z. Hu, Y. Zhu, L. Han, *Adv. Energy Mater.* **2019**, *9*, 1900896.

[15] H. Bian, D. Bai, Z. Jin, K. Wang, L. Liang, H. Wang, J. Zhang, Q. Wang, S. Liu, *Joule* **2018**, *2*, 1500.

[16] D. Bai, H. Bian, Z. Jin, H. Wang, L. Meng, Q. Wang, S. Liu, *Nano Energy* **2018**, *52*, 408.

[17] S. Yang, H. Zhao, Y. Han, C. Duan, Z. Liu, S. F. Liu, *Small* **2019**, *15*, 1904387.

[18] H. Zhao, Y. Han, Z. Xu, C. Duan, S. Yang, S. Yuan, Z. Yang, Z. Liu, S. Liu, *Adv. Energy Mater.* **2019**, *9*, 1902279.

[19] W. Chen, H. Chen, G. Xu, R. Xue, S. Wang, Y. Li, Y. Li, *Joule* **2019**, *3*, 191.

[20] K.-L. Wang, R. Wang, Z.-K. Wang, M. Li, Y. Zhang, H. Ma, L.-S. Liao, Y. Yang, *Nano Lett.* **2019**, *19*, 5176.

[21] C. F. J. Lau, M. Zhang, X. Deng, J. Zheng, J. Bing, Q. Ma, J. Kim, L. Hu, M. A. Green, S. Huang, A. Ho-Baillie, *ACS Energy Lett.* **2017**, *2*, 2319.

[22] L. Fu, Y. Nie, B. Li, N. Li, B. Cao, L. Yin, *Sol. RRL* **2019**, *3*, 1900233.

[23] W. Xiang, Z. Wang, D. J. Kubicki, W. Tress, J. Luo, D. Prochowicz, S. Akin, L. Emsley, J. Zhou, G. Dietler, M. Grätzel, A. Hagfeldt, *Joule* **2019**, *3*, 205.

[24] W. Xiang, Z. Wang, D. J. Kubicki, X. Wang, W. Tress, J. Luo, J. Zhang, A. Hofstetter, L. Zhang, L. Emsley, M. Grätzel, A. Hagfeldt, *Nat. Commun.* **2019**, *10*, 4686.

[25] S. S. Mali, J. V. Patil, C. K. Hong, *Nano Lett.* **2019**, *19*, 6213.

[26] Z. Guo, S. Zhao, A. Liu, Y. Kamata, S. Teo, S. Yang, Z. Xu, S. Hayase, T. Ma, *ACS Appl. Mater. Interfaces* **2019**, *11*, 19994.

[27] Z. Ye, J. Zhou, J. Hou, F. Deng, Y.-Z. Zheng, X. Tao, *Sol. RRL* **2019**, *3*, 1900109.

[28] Z. Wang, A. K. Baranwal, M. A. Kamarudin, Y. Kamata, C. huey Ng, M. Pandey, T. Ma, S. Hayase, *J. Mater. Chem. A* **2019**, *7*, 20390.

[29] L. Zhou, X. Guo, Z. Lin, J. Ma, J. Su, Z. Hu, C. Zhang, S. 0Liu, J. Chang, Y. Hao, *Nano Energy* **2019**, *60*, 583.

[30] Z. Wang, A. K. Baranwal, M. Akmal kamarudin, P. Zhang, G. Kapil, T. Ma, S. Hayase, *Nano Energy* **2019**, *66*, 104180.

[31] Q. Guo, Y. Bai, K. Lang, Z. Yu, T. Hayat, A. Alsaedi, E. Zhou, Z. Tan, *ACS Appl. Mater. Interfaces* **2019**, *11*, 37991.

[32] J. Zhuang, Y. Wei, Y. Luan, N. Chen, P. Mao, S. Cao, J. Wang, *Nanoscale* **2019**, *11*, 14553.

[33] S. Liu, Z. Li, Y. Yang, X. Wang, Y. Chen, D. Xue, J. Hu, *J. Am. Chem. Soc.* **2019**, *141*, 18075.

[34] W. Xu, F. He, M. Zhang, P. Nie, S. Zhang, C. Zhao, R. Luo, J. Li, X. Zhang, S. Zhao, W. Li, F. Kang, C. Nan, G. Wei, *ACS Energy Lett.* **2019**, *4*, 2491.

[35] Y. Zhang, C. Wu, D. Wang, Z. Zhang, X. Qi, N. Zhu, G. Liu, X. Li, H. Hu, Z. Chen, L. Xiao, B. Qu, *Sol. RRL* **2019**, *3*, 1900254.

[36] J. Xue, R. Wang, K.-L. Wang, Z.-K. Wang, I. Yavuz, Y. Wang, Y. Yang, X. Gao, T. Huang, S. Nuryyeva, J.-W. Lee, Y. Duan, L.-S. Liao, R. Kaner, Y. Yang, *J. Am. Chem. Soc.* **2019**, *141*, 13948.

[37] J. Tian, Q. Xue, X. Tang, Y. Chen, N. Li, Z. Hu, T. Shi, X. Wang, F. Huang, C. J. Brabec, H. Yip, Y. Cao, *Adv. Mater.* **2019**, *31*, 1901152.

[38] Z. Li, R. Wang, J. Xue, X. Xing, C. Yu, T. Huang, J. Chu, K.-L. Wang, C. Dong, Z. Wei, Y. Zhao, Z.-K. Wang, Y. Yang, *J. Am. Chem. Soc.* **2019**, *141*, 17610.

[39] L. Yan, Q. Xue, M. Liu, Z. Zhu, J. Tian, Z. Li, Z. Chen, Z. Chen, H. Yan, H.-L. Yip, Y. Cao, *Adv. Mater.* **2018**, *30*, 1802509.

[40] Q. Xiao, J. Tian, Q. Xue, J. Wang, B. Xiong, M. Han, Z. Li, Z. Zhu, H.-L. Yip, Z. Li, *Angew. Chem. Int. Ed.* **2019**, *58*, 17724.
